# Biodegradable Polyurethanes for Tissue Engineering: Influence of L-Lactide Content on Degradation and Mechanical Properties

**DOI:** 10.3390/polym17121685

**Published:** 2025-06-17

**Authors:** Alejandra Rubio Hernández-Sampelayo, Laura Diñeiro, Dulce María González-García, Enrique Martínez Campos, Rodrigo Navarro, Ángel Marcos-Fernández

**Affiliations:** 1 Institute of Polymer Science and Technology (CSIC), Juan de la Cierva 3, 28006 Madrid, Spain; alejandra@ictp.csic.es (A.R.H.-S.); ldineiro@ictp.csic.es (L.D.); e.martinez.campos@csic.es (E.M.C.); 2 Facultad de Ciencias, Universidad Nacional de Educación a Distancia (UNED), C/Bravo Murillo 38, 28015 Madrid, Spain; 3 Instituto Politécnico Nacional, Escuela Superior de Ingeniería Química e Industrias Extractivas, UPALM-Zacatenco, Col. Lindavista, Mexico City 07738, Mexico; dgonzalezg@ipn.mx

**Keywords:** biodegradability, L-lactide content, thermoplastic polyurethane, cell adhesion, hydrolysable chain extender

## Abstract

The influence of L-lactide content (between 15% and 43%) on the degradation of biodegradable polyurethanes (PUs) for tissue engineering was systematically addressed in this study. An ideal tissue scaffold should exhibit a mechanical response and degradability appropriate for the host tissue. To achieve it, polyols containing ε-caprolactone and L-lactide moieties were used, with the random distribution of lactide units disrupting the regularity, and hence the crystallinity, of poly(caprolactone) segments, facilitating their degradation. The biodegradable PUs were synthesised using these copolymers as soft segments and were characterised through various physicochemical techniques, including bioassays and water absorption measurements. It was determined that mechanical behaviour and water absorption depended significantly on molecular weight, L-lactide content in the soft segment, and the crystallinity of the hard segment. Additionally, two types of chain extenders were also evaluated: hydrolysable and non-hydrolysable. PUs based on hydrolysable chain extenders achieved higher molecular weights and exhibited better mechanical performance than their non-hydrolysable counterparts. To assess the cytocompatibility of these materials, an endothelial model was used, involving metabolic activity and DNA content analysis. The results demonstrated good cell adhesion and the absence of toxicity, confirming the viability of cell growth on the surfaces of these biodegradable PUs. The PUs developed in this study exhibited a low initial modulus and adjustable mechanical properties, highlighting their potential application in tissue engineering as biodegradable and biocompatible biomedical materials.

## 1. Introduction

Biodegradable scaffolds have emerged as a key macromolecular structure in the biomedical sector, especially in tissue engineering applications and treatments of diseases related to cell regeneration [1]. These scaffolds provide the mechanical strength needed to support cell growth and tissue formation during the healing or regeneration process, playing a crucial role in bone injury repair, cartilage regeneration, or even in the treatment of chronic wounds. A significant added value of biodegradable scaffolds is their ability to break down in a controlled manner in the body, eliminating the need for a second surgical intervention for removal. This property not only improves patient comfort but also minimises the risks associated with postoperative complications, making these materials an ideal choice for minimally invasive therapies and long-term treatments.

Despite the advantages of biodegradable scaffolds, there are several limitations that hinder their implementation in biomedical applications [2]. One of the main challenges in the development of biodegradable scaffolds is achieving precise control over the degradation rate of the material, as inadequately regulated degradation can compromise both the efficacy of the treatment and the integrity of the developing tissue. If degradation occurs too rapidly, the material breaks down before the tissue has attained sufficient stability and strength, thereby failing to provide the necessary mechanical support during the maturation phase [3]. Conversely, if degradation is too slow, the scaffold may remain functional for an excessive duration, potentially preventing complete tissue regeneration. In both scenarios, there is a risk of treatment failure—either due to premature loss of support or interference with tissue integration. Therefore, it is essential to optimise the degradation rate to ensure that the scaffold maintains adequate mechanical stability throughout the regenerative process while degrading at a pace that promotes complete tissue integration without disrupting the healing process [4,5]. In addition, many of the currently available materials fail to meet the high mechanical demands that certain applications require, such as scaffolds for bone or cartilage regeneration, where demands for strength and elasticity are critical. The challenge lies in developing materials that are not only biodegradable and biocompatible but also offer the mechanical performance needed to withstand physiological loads during the healing and regeneration process, without compromising functionality or patient safety.

Tissue engineering is defined as an interdisciplinary field of knowledge that applies the principles of engineering and health sciences to the development of biological substitutes that restore, maintain, or improve the functions of the target tissue [6]. For this aim, a combination of different elements is applied, constituting the classic triad of tissue engineering: cells, biological factors, and materials. Nowadays, several challenges should be addressed [7], specially engineering the architecture of tailored tissue scaffolds.

Polyurethanes are emerging as highly promising materials to overcome the current limitations of biodegradable scaffolds due to their extraordinary versatility and the ability to modulate their properties according to the specific requirements of each biomedical application [8]. One of their main advantages lies in the fact that their degradability is mostly concentrated in the soft segment, which allows for a finer control of the hydrolysis rate according to the physiological environment and the desired function [9]. To optimise this characteristic, different types of polyesters, such as poly(ε-caprolactone) (PCL), polylactic acid (PLA), and polyglycolic acid (PGA), have been incorporated, each providing different degradation profiles and mechanical properties [10]. Among these, PCL has been the most widely used, thanks to its recognised biocompatibility and controlled degradability. However, despite its widespread use, PCL has inherent limitations that restrict its effectiveness as a soft segment in polyurethanes for biomedical applications. Its semi-crystalline nature confers significant stiffness to the material, compromising its elastomeric behaviour, an essential property in applications requiring flexibility, deformability, and an adaptive mechanical response. This structural stiffness also has a negative impact on the degradability of the material, as the high crystallinity decreases water accessibility, slowing the hydrolysis of ester bonds and extending scaffold permanence beyond what is clinically desirable. Thus, the combination of low elasticity and slow degradation limits its applicability in scenarios where a dynamic mechanical response synchronised with tissue regeneration is needed [9].

To overcome these issues, a widely employed strategy has been the synthesis of triblock copolymers, whose design aims to improve the toughness of the material and increase its degradation rate. In this type of structure, the introduction of a second polymeric block interrupts the crystallisation of the PCL segment, reducing its structural order and favouring degradation. Copolymers based on polyethylene glycol (PEG) [11], polytetramethylene glycol (PTMG) [12], or polypropylene glycol (PPG) [13] have been explored with positive results. However, this approach has an important limitation; triblock polyols usually have molecular weights above 2000 g/mol, which makes it difficult to achieve the optimal elastomeric properties observed in polyurethanes formulated with polyols with molecular weights between 1000 and 2000 g/mol [14]. As a result, these materials fail to provide the desired elasticity and recovery under repeated stresses that are essential for temporary support applications. Faced with this challenge, a more promising alternative is to randomly introduce a second co-monomer into the polyol chain. This disruptive architecture hinders the crystallisation of the PCL segment while maintaining molecular weights within the ideal range for elastomeric behaviour. This strategy not only improves the flexibility of the material but also promotes faster and more homogeneous degradation of the soft segment. Although such PCL-PLA random polyols are already commercially available, no systematic study has been carried out so far to analyse how the PLA ratio and the polyol molecular weight jointly influence the mechanical properties and degradability of the resulting polyurethanes.

The present work deals with the design and development of polyurethanes from polycaprolactone-derived polyols functionalized with L-lactide, evaluating their thermal, mechanical, and biodegradation properties. The incorporation of L-lactide into the soft segments led to a reduction in crystallisation, which favoured the hydrolytic and/or enzymatic degradation of the polyurethanes. In addition, another series of biodegradable polyurethanes was prepared in which the hard segment was also hydrolysable, using EDA-2CL as a chain extender. This approach allowed for both segments (hard and soft) to be degraded more easily, shortening the biodegradation times. The ability to modulate the degradation rate, on the one hand, allows for the creation of highly versatile systems, adapting to the specific needs of each application, and, on the other hand, facilitates the development of sustainable materials that degrade at the end of their useful life, avoiding their undesired accumulation in the body. Ultimately, this approach not only improves the functionality of materials in different contexts but also ensures their controlled degradation, promoting efficient biodegradation.

## 2. Materials and Methods

### 2.1. Materials

1,4-Butanediol (BD), tin(II) 2-ethylhexanoate (Sn(Oct)_2_) and 1,1,1,3,3,3-hexafluoro-2-propanol (HFIP) were purchased from Sigma-Aldrich (Madrid, Spain) and used as received. Aliphatic isocyanate 1,6-hexamethylenediisocyanate (HDI) was purified by distillation under reduced pressure. Dimethylacetamide (DMAc) was purified by reduced-pressure distillation in the presence of a commercial polymeric MDI-isocyanate to remove traces of water or amines.

Commercial polycaprolactone diols (CAPA^®^ series 8000) with nominal molecular weights of 1000, 2000, and 3000 g/mol, were kindly supplied by Perstorp (Warrington, UK). The exact molecular weights of the CAPA^®^ diols were determined by proton NMR [14,15], yielding values of 1017 (CAPA^®^-8015), 1973 (CAPA^®^-8021), 1970 (CAPA^®^-8025), and 2987 (CAPA^®^-8038) g/mol. All polyols were dried under vacuum at 70 °C for 3 h and stored in a vacuum desiccator until use.

### 2.2. Characterisation

Proton Nuclear Magnetic Resonance (NMR) spectra were recorded on a Varian Unity Plus 400 spectrometer (Palo Alto, CA, USA), at room temperature, using deuterated chloroform (CDCl_3_) or deuterated DMSO as solvent. NMR spectra were referenced to the residual solvent peak at 7.26 ppm for chloroform-d and at 2.50 ppm for DMSO-d_6_.

The number and weight-average molecular weights of polyurethanes were measured by size exclusion chromatography (SEC) using a Water apparatus (Waters Division Millipore, Madrid, Spain), equipped with a refractive index detector. A set of Styragel HR3 and HR5 Waters columns (300 × 7.8 mm, 5 mm nominal particle size) conditioned at 70 °C was used to elute the polymer solutions at a 0.7 mL/min flow rate. The mobile phase was *N*,*N*-dimethylformamide (DMF) with 0.1% of LiBr. Polystyrene standards (Polymer Laboratories) were used to calibrate the system.

Fourier-transform infrared (FTIR) spectra were acquired using a Perkin-Elmer spectrometer, model Spectrum One (Perkin-Elmer, Waltham, MA, USA), equipped with an attenuated total reflectance (ATR) accessory. Each spectrum represents the average of 16 scans recorded over the range of 4000 to 450 cm^−1^ with a resolution of 2 cm^−1^.

Thermogravimetric analysis (TGA) was performed using a Mettler Toledo TGA/SDTA 851 instrument (Mettler-Toledo, Schwerzenbach, Switzerland). The polyurethane films were heated from room temperature to 600 °C under a nitrogen atmosphere at a heating rate of 10 °C/min.

Differential scanning calorimetry (DSC) was carried out in a Mettler Toledo 822 e calorimeter equipped with a liquid nitrogen accessory. Small polyurethane discs (10–15 mg) were weighed into aluminium pans. The thermal treatment consisted of an initial heating from room temperature to 200 °C, followed by controlled cooling to −90 °C. The sample was then held at −90 °C for 5 min and then reheated from −90 to 200 °C. In all steps, the heating/cooling rate was 10 °C/min. The glass transition temperature (Tg) was determined as the midpoint of the heat capacity change during transition.

Mechanical properties were measured using an MTS Synergie 200 testing machine equipped with a 100 N load cell. Test specimens were cut according to ISO 37 [16] (Type 4) dimensions. Tensile tests were carried out at a crosshead rate of 5 mm/min, and the strain was measured from an initial gauge length of 10 mm. For each polyurethane sample, a minimum of five specimens were tested.

### 2.3. Synthesis of N-N’-Ethylene-bis(6-hydroxycaproamide) (EDA-2CL)

**Scheme 1:**
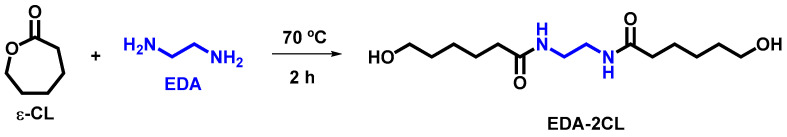
Synthetic route for *N*,*N*′-ethylene-bis(6-hydroxycaproamide).

The synthesis of this chain extender was carried out according to the synthetic route outlined in Scheme 1 and following the synthetic steps described elsewhere [17,18]. The procedure involved heating 0.2 mol of 1,2-ethylenediamine (EDA) and a large excess of e-caprolactone (2.0 mol) at 70 °C for 2 h. After the reaction mixture cooled, the desired product precipitated as a white solid, which was isolated by filtration and washed with toluene.

### 2.4. Synthesis of Segmented Poly(Ester-Urethane)s

Two sets of segmented polyurethanes were synthesised, differing in hard segment content, one with 35% and the other with 50%, defined as (weight of HDI + chain extender)/total weight. The synthesis followed a two-step procedure. In the first step, HDI and the polyol (CAPA^®^) were weighed in the appropriate stoichiometric ratio and dissolved in *N*,*N*-dimethylacetamide (DMAc) at a 50:50 (*w*/*v*) ratio. The reaction was catalysed using tin octoate (Sn(Oct)_2_) and carried out at 80 °C for 3 h under a continuous flow of nitrogen. In the second step, the corresponding amount of chain extender—either 1,4-butanediol (BD) or (EDA-2CL)—was added, and the reaction mixture was maintained at 80 °C for an additional 3 h, followed by reaction at room temperature overnight. To isolate the segmented polyurethane, the viscous solution was cast onto a levelled heating plate and allowed to evaporate slowly at 60 °C to remove the solvent. Upon complete solvent removal, a homogeneous film was obtained and stored under vacuum until further use.

### 2.5. Hydrolytic and Enzymatic In Vitro Degradation

Multiple polyurethane samples were immersed in a phosphate-buffered saline (PBS) solution (pH = 7.4) and incubated at 37 °C without solution renovation. A defined quantity of each film was placed in individual vials, and in vitro degradation was assessed by monitoring changes in the hydrated mass of the polymer films at specific time intervals. At certain time intervals, the films were removed, blotted with filter paper, immediately weighted with an analytical balance, and returned to their respective vials. After the polymers had been degraded, the films were washed off and dried in a vacuum oven.

The difference in mass of each sample resulting from the water uptake was calculated according to the following Formula (1) and indicated as a percentage:

Water uptake (%) = ((W_m_ − W_0_)/W_0_) × 100 (1)

where W_m_ is the weight of the swollen specimen and W_0_ is the initial weight of the sample.

Additionally, the samples were removed, exhaustively dried under vacuum, and weighed at specific time frames. The weight loss percentage was calculated from the following Equation (2):

Weight loss (%) = ((W_0_ − W_t_)/W_0_) × 100 (2)

where W_t_ is the weight of the dried specimen at a determined time t.

Enzymatic degradation was assessed using porcine liver esterase (PLE) (Sigma-Aldrich, Madrid, Spain) prepared at a concentration of 30 U/mL in Dulbecco’s PBS. The enzymatic solutions were sterilised by filtration through 0.22 µm filters. To ensure constant enzymatic activity, the PLE solution was refreshed every 24 h. The weight variation in the samples was recorded using Equation (2), and all enzymatic degradation experiments were conducted in triplicate.

### 2.6. Electrospinning

A polyurethane solution of PU8038-HB-50 (5 mL of a 10 wt% solution in HFIP) was electrospun onto rigid glass supports to form fibre meshes. After testing several concentrations, this concentration was selected to obtain meshes composed of uniformly shaped fibres. The electrospinning was performed under ambient conditions using a 22-gauge Teflon-coated needle, an applied voltage of 19 kV, a tip-to-collector distance of 14 cm, and a syringe flow rate of 500 µL/h using a Model Fluidnatek LE-50, Valencia, Spain. The meshes were dried and stored in a desiccator to remove residual HFIP and to minimise degradation until use.

### 2.7. Scanning Electron Microscopy

A scanning electron microscope (SEM) (SNE-Alpha Tabletop-SEM), Thermo Scientific, Waltham, MA, USA was employed to examine the morphology and thickness of the electrospun fibres. For this purpose, each mesh was coated with a thin layer of gold prior to scanning electron microscopy.

### 2.8. Cytocompatibility Evaluation of Polyurethane Samples

Prior to cell culture, electrospun polyurethanes meshes and cast films were placed in a 24-well tissue culture plate (Fisher Scientific, Waltham, MA, USA) and subjected to three consecutive washes in phosphate-buffered saline (PBS; Thermo Fisher, Waltham, MA, USA), each lasting 10 min. Then, the samples were sterilised by exposure to ultraviolet (UV) light for 40 min (20 min per side). Following sterilisation, each sample was covered with 3 mL of Dulbecco’s Modified Eagle Medium (DMEM 1×; Gibco, Waltham, MA, USA), supplemented with 10% foetal bovine serum (FBS; Thermo Scientific, Waltham, MA, USA) and antibiotics (100 U/mL penicillin and 100 μg/mL streptomycin sulphate; Sigma-Aldrich, St. Louis, MO, USA).

Cell viability and proliferation assays were conducted using the C166-GFP autofluorescent mouse endothelial cell line (ATCC CRL-2583^TM^, ATCC, Manassas, VA, USA). A total of 20,000 cells per sample were seeded onto the polyurethane films and electrospun meshes and cultured for 8 days. Cell attachment and proliferation were monitored daily using inverted fluorescence microscopy (Olympus IX51, Tokyo, Japan) equipped with an FITC filter (λex/λem = 490/525 nm).

After 8 days, endothelial cell metabolic activity was assessed using the Alamar Blue assay (Biosource), a non-toxic method that exploits the intrinsic reducing capacity of viable cells to provide a quantitative measure of cell viability and cytotoxicity. Briefly, Alamar Blue reagent was added to each well at 10% of the culture medium volume, and the samples were incubated for 90 min. Fluorescence intensity was then measured using a Synergy HT plate reader (BioTek, Winooski, VT, USA) at 535/590 nm.

Statistical analyses were conducted using Student’s *t*-test with GraphPad Prism software (version 10.4.0). Significant differences represent * (*p* ≤ 0.05).

## 3. Results and Discussion

The selection of CAPA^®^ polyesters from the 8000 series as raw materials for polyurethane synthesis was driven by their unique chemical composition and tuneable properties. These polyols are random copolyesters composed of ε-caprolactone (ε-CL) and lactic acid (LA) units, with variable LA content. This structural configuration enables the fine-tuning of physicochemical characteristics to meet the specific demands of biomedical or industrial applications. Their molecular weights are well-suited for the synthesis of high-performance polyurethane elastomers, as they combine the flexibility and processability of PCL with the increased rigidity and biodegradability associated with PLA. Moreover, the random incorporation of PLA units disrupts the crystallinity of PCL, thereby enhancing the susceptibility of the material to hydrolytic degradation.

**Scheme 2:**
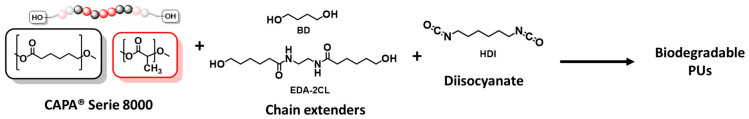
Synthetic route for the preparation of biodegradable poly(ester-urethane)s.

In this study, four PCL-based polyols from the CAPA^®^ 8000 series (CAPA 8015, 8021, 8025, and 8038) were used as soft segments for the preparation of biodegradable linear polyurethanes. Hexamethylene diisocyanate (HDI) served as the diisocyanate component, while two types of chain extenders—BD and EDA-2CL—were incorporated to evaluate the influence of hydrolytically stable and degradable extenders, respectively. The NCO:OH molar ratio was maintained at a strict stoichiometric balance in all formulations to promote the formation of high-molecular-weight linear polymers (Scheme 2).

The synthesised poly(ester-urethane)s were fully soluble in polar aprotic solvents such as *N*,*N*-dimethylacetamide (DMAc), *N*,*N*-dimethylformamide (DMF), dimethyl sulfoxide (DMSO), and hexafluoroisopropanol (HFIP), facilitating both processing and characterisation. For clarity, the samples were designated using the code PUX-HY-Z, where X corresponds to the CAPA^®^ polyol used (8015, 8021, 8025 or 8038), H denotes the use of HDI, Y indicates the chain extender (B for BD, E for EDA-2CL), and Z refers to the hard segment content (35 or 50 wt%).

The following sections detail the characterisation of the synthesised polyurethanes. Two chain extenders with distinct hydrolytic behaviours were selected to study their impact on polymer properties. Additionally, the hard segment content was varied between 35% and 50% to assess its influence on the structural, thermal, mechanical, and degradation profiles of the resulting materials.

### 3.1. ^1^H-NMR Data

**Figure 1:**
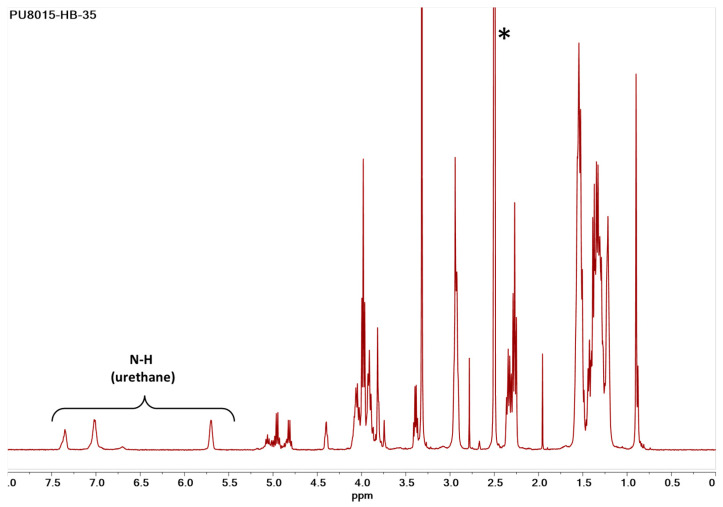
^1^H-NMR spectrum of poly(ester-urethane) PU8015-HB-35 in deuterated dimethylsulfoxide (DMSO-d6). The residual peak of the deuterated solvent is indicated by an asterisk (*).

The chemical structure of synthetised polyurethanes bearing L-lactide-based polyols were analysed by ^1^H-NMR spectroscopy. In Figure 1, the ^1^H-NMR spectrum of poly(ester-urethane) (PU8015-HB-35) derived from the reaction of CAPA^®^8015, HDI, and BD is depicted. The characteristic signals of methylenes close to caprolactone moiety [-*CH_2_*-O-(C=O)-, d 3.97 ppm] and the methine group [-*CH*-O-(C=O)-, d 4.99 ppm] demonstrated that the random copolymer PCL-PLA could be easily introduced in the main chain of the PU. The rest of the peaks were assigned based on research data [19,20]. Moreover, the absence of the isocyanate group in the spectrum confirmed the completion of the reaction, demonstrating that the stoichiometric ratio of the three reactants was accurately calculated and effectively maintained throughout the synthesis.

### 3.2. Mechanical Properties and Molecular Weight

In the development of new biodegradable polyurethanes, evaluating their molecular weight and mechanical properties not only provides essential information about their microstructure and composition but also offers a comprehensive view of their potential functional performance. Unlike other characterisation techniques, these measurements allow for direct correlations between the macromolecular architecture of the polymer and its response to physical stress, which is crucial when designing materials with specific requirements for strength, flexibility, or durability (especially in relation to biodegradability). In particular, knowing the molecular weight helps to anticipate the behaviour during processing, while mechanical testing reveals the viability of the material under real-world conditions. The molecular weights and mechanical response of biodegradable polyurethanes are listed in Table 1.

The molecular weights (Mn) determined by gel permeation chromatography (GPC) were remarkably high in almost all cases, between 93 and 255 kDa (except for PU8015-HB-50 and PU8025-HB-50, with Mn below 40 kDa). This outcome reflects the high degree of control during the polymerisation reaction, particularly due to the precise adjustment of the stoichiometric balance between isocyanate (R-NCO) and hydroxyl functional groups (R’-OH), which promoted efficient and extensive chain growth. Another key factor was the accurate determination of the molecular weight of the CAPA^®^ polyols, which enabled the precise calculation of the required amount of each reactant to maintain stoichiometric balance, thereby reducing the presence of unreactive terminal groups. Additionally, the solvent used for the polymerisation—*N*,*N*-Dimethylacetamide (DMA)—was thoroughly dried, minimising undesired side reactions such as isocyanate hydrolysis, which could have limited the molecular weight of the final polyurethane. It is well known that traces of water can react with isocyanate groups, altering the NCO:OH stoichiometric ratio (1:1) and hindering efficient polyurethane chain growth [21,22]. High molecular weights are necessary to obtain materials with superior mechanical performance, as it is well established that in polyurethanes (and in polymers in general), higher molar mass correlates with improved mechanical properties such as tensile strength and elasticity [23].

As shown in Table 1, the mechanical tests revealed that, within the series using 1,4-butanediol (BD) as a chain extender, the highest maximum strength was attained at a hard segment content of 50%. However, this increase in strength was accompanied by a decrease in elongation at break, which was found to be lower under these conditions. For the polymers PU8015-HB-50 and PU8025-HB-50, the low molecular weight (< 40 kDa) was reflected in materials with low elongation at break.

The influence of the polyol molecular weight on the mechanical properties was analysed by comparing pairs of polyols with similar lactide contents but different molar masses. In the first case, CAPA^®^ 8015 and CAPA^®^ 8025 polyols (14% and 18% lactide, respectively) were compared. It was observed that the latter, with a Mn of 2000 g/mol, generated a higher maximum stress in tensile tests. This effect was attributed to a higher tendency to crystallisation induced during deformation. Equivalent behaviour was identified when comparing the CAPA^®^ 8021 and CAPA^®^ 8038 polyols, with lactide contents of 43% and 37.9% and molecular weights of 2000 and 3000 g/mol, respectively. In this second pair, the higher Mn polyol also demonstrated superior mechanical performance, presumably for the same reason. These results suggest that, under constant PLA content, an increase in the molecular weight of the soft segment favours its ability to crystallise under stress, resulting in higher mechanical strength. This hypothesis will be further explored in the section devoted to thermal analysis by DSC.

Finally, the mechanical behaviour of the series synthesised with the hydrolysable chain extender EDA-2CL was evaluated. The findings demonstrated that this chain extender resulted in a substantial enhancement of the mechanical properties when the hard segment content was 35%. In particular, a remarkable increase in the maximum strain was recorded, exceeding the values obtained with the traditional non-hydrolysable extender (BD). This enhancement was particularly evident in the systems utilising polyols CAPA^®^ 8015 and CAPA^®^ 8038, where the maximum stress was almost doubled in comparison to their counterparts in the BD series. The strain at break exhibited a similar trend, indicating that the use of EDA-2CL not only increases the mechanical strength but also enhances the ductility of the material. These results underline the potential of EDA-2CL in the formulation of high-performance polyurethanes with adjustable characteristics according to application requirements.

All experimental stress–strain curves from the tensile tests of the different biodegradable polyurethanes studied are provided in the Supplementary Materials.

### 3.3. Thermal Properties

Beyond mechanical performance, the thermal responses of polymeric materials provide critical information on their heat stability, processing behaviour, and resistance to thermal degradation. These characteristics allow for anticipating the performance of the material in demanding environments or under industrial conditions. In this section, results obtained by thermogravimetry (TGA) and differential scanning calorimetry (DSC), techniques that together provide complementary insights into the thermal resistance and key physicochemical transitions of the developed polyurethanes, are discussed.

#### 3.3.1. Thermogravimetric Analysis (TGA)

TGA is an essential tool for assessing the thermal stability of polymeric materials, as it provides crucial information on the decomposition processes, degradation temperatures, and thermal resistance of the material. The results obtained from the TGA tests showed that all the polyurethanes studied lost almost 95% of their weight in a single drop, suggesting that the decomposition of the soft segment occured in an overlapping manner with that of the hard segment. In previous works, it was shown that polyurethanes bearing pure PCL polyols showed two stages of mass loss, the first associated with the decomposition of the urethane groups and the second linked to the decomposition of the PCL ester in the hard segment. However, in these new polyurethanes, the thermal decomposition of the PLA units probably occurs at an intermediate temperature between the urethane and the PCL ester. This behaviour is in line with the thermal behaviour of the starting polyesters, since PLA has a slightly lower thermal stability than its PCL counterpart [24].

#### 3.3.2. Differential Scanning Calorimetry (DSC)

The measurement of thermal transitions by DSC is a fundamental step in the characterisation of polymers, as it enables the identification of key changes in their physical structure, such as the glass transition temperature (Tg), melting temperature (Tm), and crystallisation temperature (Tc). These thermal parameters are critical for understanding the thermal behaviour, stability, and processability of the polymer, among other performance aspects.

In this study, the thermal transitions of the polyurethanes were determined by conducting two controlled heating scans at a rate of 10 °C/min, allowing for precise identification of events such as glass transition, melting, and potential structural reorganisation processes. Between these two heating cycles, a controlled cooling scan was performed at a rate of −10 °C/min to investigate the crystallisation behaviour of the polyurethanes under defined conditions. This methodology enabled not only the assessment of the thermal reproducibility of the polymers but also an evaluation of how thermal history influences their behaviour, providing critical insight into their stability and processing performance. Table 2 presents the Tg and Tm values and melting enthalpies (ΔH_f_) form the second heating cycle for all synthesised polyurethanes.

In all cases, the glass transition temperatures of the soft segments were found to be well below ambient temperature. Given that the Tg of pure PCL is approximately −60 °C and the Tg of pure PLA is around +60 °C, it would be expected that increasing the content of the renewable polyester (PLA) would progressively raise the Tg of the soft segments. It was observed that even in the polyol with the highest PLA content (CAPA^®^-8038, with 43% of PLA), the Tg remained at −11.8 °C. This suggests a particular compatibility between the two components or a non-uniform distribution of PLA moieties along the chain, which prevents the formation of sufficiently large rigid domains capable of significantly impacting the thermal behaviour of the soft segment. As a result, the Tg could remain primarily governed by the high flexibility of the PCL units.

A more detailed analysis of the variation in glass transition temperature (Tg) among the different polyurethanes revealed key structure–property relationships. In the series synthesised using 1,4-butanediol (BD) as a chain extender, it was observed that the Tg of the soft segment increased with a higher hard segment (HS) content. In principle, the Tg values of polyurethanes bearing 35% HS should be lower than their 50% HS counterparts, since a higher fraction of hard segments restricts chain mobility. However, this behaviour effectively attenuates as the molecular weight of the CAPA^®^ polyol increases.

Moreover, a deeper examination of the Tg values revealed a positive correlation with the PLA content in the soft segment; as the proportion of PLA increased, so did the Tg. This trend is attributable to the higher intrinsic rigidity of LA units compared to its polyester counterpart PCL. However, the number-average molecular weight (Mn) of the soft segment must also be considered, as an increase in Mn was found to correlate with a decrease in Tg when the PLA and HS contents were held constant. This was evident when comparing the polyols CAPA^®^-8015 and CAPA^®^-8025, which have Mn values of approximately 1000 and 2000 g/mol, respectively, and Tg values of −36.8 °C and −43.8 °C. This trend could be explained by the fact that in soft segments with lower Mn (e.g., CAPA^®^-8015), the relative density of urethane groups is higher, leading to stronger dipole–dipole interactions and increased local chain rigidity, thereby reducing segmental mobility and elevating the Tg of the system [10]. These combined factors—chemical composition and molecular weight—highlight the complex interplay that governs thermal behaviour in segmented polyurethane systems.

Regarding the melting enthalpies (ΔH_f_), a progressive increase was observed as the hard segment (HS) content in the formulations increased. This behaviour was expected, as a higher proportion of hard segment promotes the formation of more extensive and defined crystalline domains, which directly contributes to the increase in melting enthalpy. Furthermore, the enhanced crystallinity of the hard segment promotes more pronounced microphase separation between soft and hard domains, thereby contributing to the development of a well-defined phase morphology [25].

A similar trend was observed when comparing the polyurethanes PU8021-HB-50 (Mn ≈ 2000 g/mol, 43% PLA) and PU8038-HB-50 (Mn ≈ 3000 g/mol, 37.9% PLA), with melting enthalpies that were markedly different—24.03 J/g for PU8021-HB-50 and 38.44 J/g for PU8038-HB-50. This difference indicates that, while the higher Mn of CAPA^®^-8038 may facilitate crystalline organisation of the longer hard segments, the elevated fraction of PLA moieties in CAPA^®^-8021 (43%) acts as a limiting factor. PLA chains, due to their rigidity, reduce the mobility required for crystallisation, particularly when present in high proportions. This behaviour highlights how not only molecular weight, but also the specific composition and distribution of units within the soft segment, critically influence the crystallisation capacity of segmented polyurethane systems.

The influence of PLA content on melting enthalpy was further evaluated by comparing polyurethanes with identical hard segment content (50%) and soft segments of similar molecular weight, specifically the polyols CAPA^®^-8021 and CAPA^®^-8025, both with Mn values around 2000 g/mol. Under these conditions, the only significant variable was the PLA content—43% in PU8021-HB-50 and 18% in PU8025-HB-50. Again, it was found that increasing the PLA proportion led to a decrease in melting enthalpy, indicating a reduced ability of the system to crystallise.

In the series of polyurethanes based on the chain extender EDA-2CL, a regular and hydrolysable motif, the glass transition temperature (Tg) values followed a trend similar to that observed for its non-hydrolysable counterpart (BD). In this case, Tg increased as the content of the soft segment (SS) was increased, as well as when the content of PLA in the soft segment was raised. Additionally, the increase in the PLA fraction, known for its rigidity, also contributed to the rise in Tg by decreasing the overall flexibility of the system. These results highlight that, although the type of chain extender (hydrolysable or non-hydrolysable) influences certain properties of the material, the thermal behaviour, especially with regard to Tg, follows a similar trend when varying the hard segment and PLA content.

**Figure 2:**
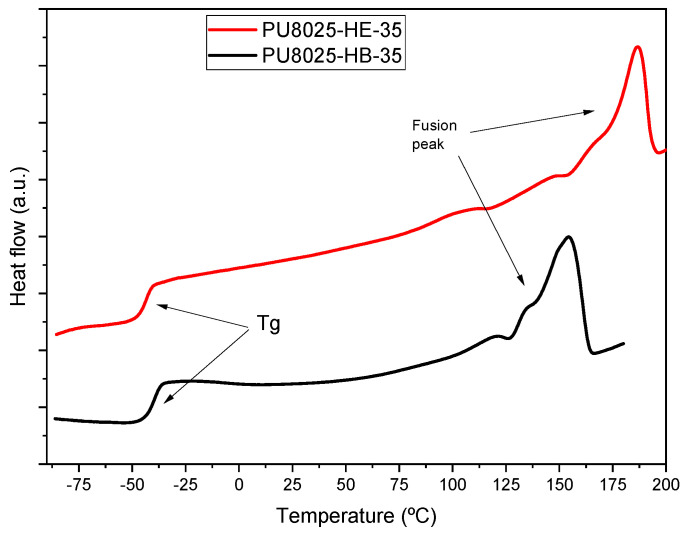
Second heating DSC thermograms of two PU samples synthetized with different chain extender.

Regarding the fusion enthalpies, it was observed that for the new series of polyurethanes based on the chain extender EDA-2CL, the values were slightly lower compared to the BD-based series. However, a noteworthy aspect was that the fusion peak of the new series appeared at significantly higher temperatures. While the fusion peaks of BD-based polyurethanes were in the temperature range of 60 to 160 °C, the fusion peaks of the EDA-2CL-based polyurethanes occurred in the range of 160 to 200 °C. (Figure 2) This difference could be attributed to the presence of highly polar amide groups in the EDA-2CL chain extender, which require more thermal energy to overcome molecular interactions and enable the fusion of the crystalline domains in the hard segments. The higher polarity of these groups could increase the rigidity of the hard segment and, therefore, raise the temperature required for fusion, resulting in fusion peaks at higher temperatures.

### 3.4. Hydrolytic and Enzymatic Degradation

Hydrolytic and enzymatic degradation assays are essential for evaluating the long-term stability and biodegradability of polymeric materials under physiological and environmental conditions. These tests provide valuable insights into the mechanisms and kinetics of material breakdown, which are critical for applications in biomedical devices, tissue engineering, and sustainable material design.

**Figure 3:**
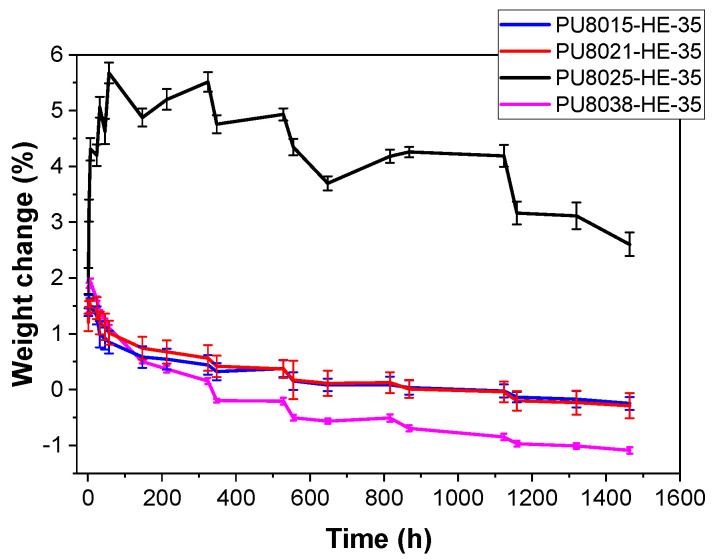
Weight variation in weight with time after the immersion in PBS for polyurethanes based on CAPA^®^-8015, CAPA^®^-8021, CAPA^®^-8025, and CAPA^®^-8038 polyols and 35% HDI-EDA-2CL as HS.

The changes in sample mass over a period of 0 to 1500 h in water are presented in Figure 3. These variations reflect the water uptake capacity of the materials and serve as an indirect indicator of their hydrophilicity. For all studied polymers, weight change curves presented two clearly differentiated states. At the beginning of the immersion, a maximum water absorption was reached, followed by a very slow decay of weight over time. The shape of the weight loss curves in the swollen state is characteristic of surface erosion, with indicating very low total degradation. As shown in Figure 3, when comparing polyurethanes synthesised from CAPA^®^-8021 and CAPA^®^-8025—which possess comparable molecular weights but differ in lactic acid content—it is evident that degradation proceeds more rapidly in the material based on the polyol with a higher lactic acid content. Specifically, the polyurethane derived from CAPA^®^-8025 (PU8025-HE-35), which has a lower lactic acid proportion, exhibits a significant initial swelling, with its mass increasing by approximately 6% within the first 100 h. Subsequently, its weight decreases progressively, following a trend similar to that observed for the other polyurethanes. The higher initial swelling for PU8025-HE-35 (circa 6% weight) could be explained by a higher amount of amorphous phase, which allows for a higher penetration of water in the material. This behaviour has also been found in a series of polyurethanes with PCL of different lengths as soft segment, where the completely amorphous PU swelled by 80% in weight despite the hydrophobicity of the PCL soft segments [26]. This higher amount of amorphous phase is difficult to demonstrate from DSC data, although this PU combines the lowest Tg (softer amorphous phase) and the second lowest hard segment crystallinity. Nevertheless, all PUs, including PU8025-HE-35, showed a high hydrophobicity with low values of water swelling (below 2% weight PU8025-HE-35 excluded).

Figure 3 also highlights the influence of polyol molecular weight on degradation behaviour when the lactic acid content is held constant. In the case of CAPA^®^-8021 versus CAPA^®^-8038, the polyurethane formulated with the higher molecular weight polyol (CAPA^®^-8038) shows a more pronounced and rapid mass loss. This could be attributed to the greater number of accessible ester bonds per repeating unit in the longer chains, which increases the rate of hydrolysis. A comparable behaviour is observed in the CAPA^®^-8015 versus CAPA^®^-8025 pair, where the polyurethane synthesised from the longer-chain polyol (CAPA^®^-8025) undergoes slightly faster degradation compared to its lower-molecular-weight counterpart.

**Figure 4:**
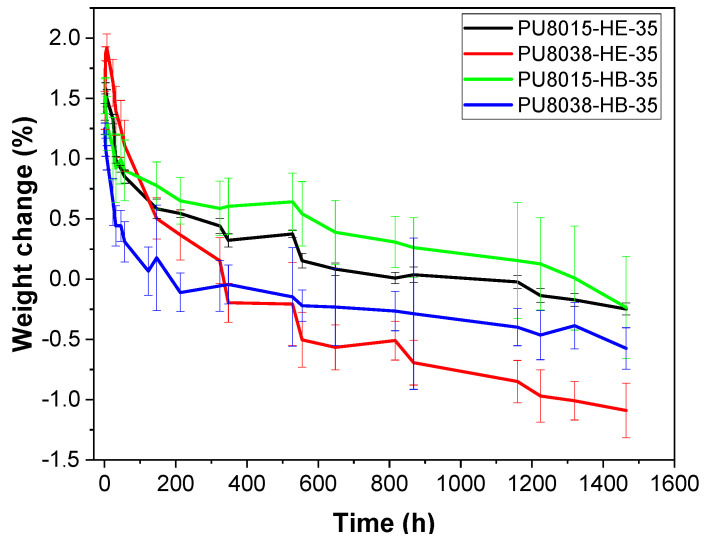
Weight variation with time after the immersion in PBS of polyurethanes based on CAPA^®^-8015, CAPA^®^-8038 polyols, HDI, and EDA-2CL or BD as HS.

The hydrolytic degradation profiles of the polyurethanes synthesised with different chain extender are presented in Figure 4, highlighting the influence of the chain extender nature on the degradation behaviour. Regarding the nature of the soft segment and chain extender, it was observed that the maximum water absorption was slightly higher for PU8038-HE-35, (1.92%) compared to its non-hydrolysable chain extender counterpart (BD) and polyol. The increase in water absorption may be due to the incorporation of PLA moieties into the polyol (SS), which led to a reduction in crystallisation and thus favoured the hydrolytic degradation of the PU, with the amorphous soft segment regions being degraded prior to the degradation of the crystalline hard segment regions. Polymer hydrolysis is partly controlled by the rate of water diffusion in the amorphous regions of the polymer, since water diffusion through crystalline regions is negligible. Some plastics, such as PCL, will not biodegrade without prior hydrolysis. The nature of the chain extender also plays an important role in the hydrolysis of the polymer, because the polar (amide) groups provided by EDA-2CL would increase hydrophilicity through hydrogen bonding with water molecules, leading to increased water adsorption. Comparing both effects, it was appreciated that the nature of the chain extender was more relevant in relation to the effect provided by the soft segment.

Biodegradation is primarily an enzymatic process and is therefore highly specific to the chemical structure and bonding present within the polymer. Biodegradable polymers typically incorporate hydrolysable bonds such as esters, amides, or carbonates within their backbone, which increases their susceptibility to degradation. Other factors that affect biodegradability are crystallinity, molecular weight, and chemical compositions [27]. Among synthetic biodegradable materials, aliphatic polyesters with hydrolysable ester linkages—such as PLA, PCL, and polybutylene succinate (PBS)—are particularly significant due to their well-documented susceptibility to both hydrolytic and enzymatic degradation [28].

The enzymatic degradation of the synthesised polymers was assessed gravimetrically using porcine liver esterase (PLE). The degradation behaviour of a polymer is fundamentally determined by its chemical structure. Polymers containing stable carbon–carbon bonds often resist biological degradation, as these bonds cannot be cleaved by enzymes or microorganisms. Furthermore, a hydrophobic character can limit enzymatic activity, particularly when combined with factors such as low surface area, high molecular weight, and a high degree of crystallinity. Table 3 summarises the weight variation in each sample after 7 and 21 days of immersion, in the presence or absence of the PLE enzyme.

As shown in Table 3 after 7 and 21 days of incubation, in the absence of an enzyme, the polyurethane samples showed a small swelling. However, in the presence of PLE, the degradation of the polyurethanes was significantly pronounced. The enzyme PLE preferentially promoted the hydrolysis of ester bonds contained in the soft segment.

An increase in the molecular weight of the polyol (CAPA) was found to accelerate the enzymatic degradation rate of the polyurethanes. In particular, the formulation based on CAPA^®^-8038 exhibited a weight loss nearly two-folds higher than its polyurethane counterparts after both 7 and 21 days of enzymatic exposure. This behaviour is consistent with the results obtained under hydrolytic conditions and was attributed to the greater number of hydrolysable functional groups, such as ester linkages, present per repeating unit in higher-molecular-weight polyols. The increased availability of these reactive sites enhances the susceptibility of the polymers to enzymatic attack.

Furthermore, the degradation data revealed that the hard segment content plays a significant role in modulating the enzymatic resistance of the material. Polyurethanes containing 50% HS displayed substantially lower weight loss compared to their 35% HS counterparts, suggesting that the hard segments are more resistant to enzymatic degradation. This observation is consistent with the more rigid and densely packed structure of the hard domains, which hinders the accessibility of enzymes to hydrolysable bonds and thus slows the degradation process.

### 3.5. Electrospinning

Electrospinning has emerged as a versatile and highly effective technique in biomedicine for the fabrication of porous polyurethane scaffolds [29]. Among the various methods available for processing polyurethane into scaffold structures, electrospinning stands out [30] for its ability to produce highly controllable, ultra-fine polymer fibres and three-dimensional architectures with morphological characteristics similar to the extracellular matrix (ECM). This technique is particularly advantageous in tissue engineering applications—including heart valve regeneration [31]—where nanofibrous scaffolds with high surface-area-to-volume ratios enhance cell adhesion, proliferation, and tissue integration. Moreover, the flexibility of the electrospinning process allows for precise control over the scaffold microstructure, including the fabrication of anisotropic fibre orientations to better mimic native tissue architecture. Electrospinning operates on the principle of applying electrostatic forces to a polymer solution, suspension, or melt to generate continuous fibres with variable diameters. The essential setup consists of a high-voltage power supply, a spinneret (typically a metallic needle), and a grounded collector [32].

**Figure 5:**
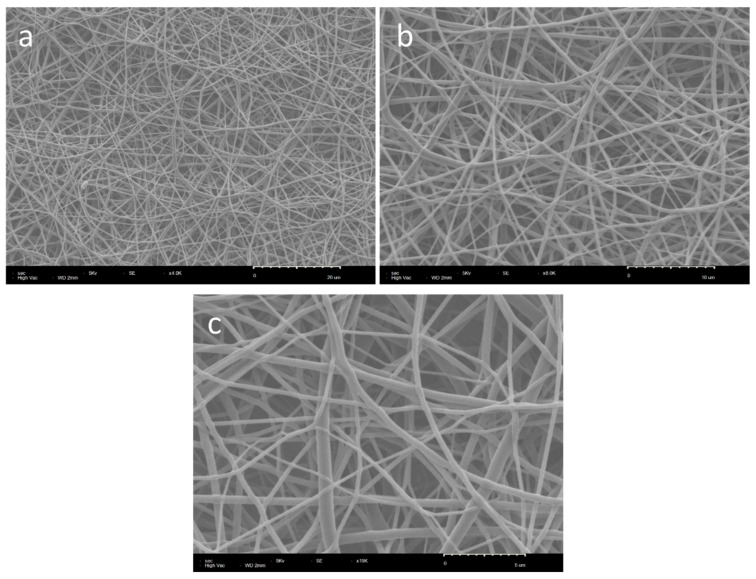
SEM micrographs of scaffold PU8038-HB-50 (**a**) ×4000 (**b**) ×8000 (**c**) ×15,000.

The scanning electron micrographs (SEM) presented in Figure 5 reveal a matrix of electrospun fibres of polyurethane (PU8038-HB-50), with diameters ranging from 350 to 990 nm. These values were obtained from a polymer solution at 10 wt%, a concentration that falls within the intermediate range reported in the literature for producing high-quality fibres [33]. The diameters achieved are consistent with the viscosity of the solution, which was made possible by the high molecular weight of the polyurethane used (255 kDa). This elevated molecular weight promotes a greater degree of chain entanglement, thereby increasing the solution viscosity and ensuring the stability of the jet during the electrospinning process [34,35,36]. It is important to note that the viscosity must reach a critical balance. If too low, the solution lacks the necessary entanglement density, leading to the formation of beads or electrosprayed droplets; if sufficient, but the chain overlap remains below the critical threshold, bead-on-string morphologies may still occur [13,37]. As described in previous studies [38,39,40], increasing the viscosity not only reduces the extent of jet stretching under the electric field, resulting in thicker fibres, but also alters the morphology of the beads—from spherical droplets at low viscosities to elongated ellipsoids and eventually to smooth, bead-free fibres as viscosity increases. Zong et al. further observed that bead spacing increases with viscosity, highlighting the complex interplay between viscoelastic properties and fibre morphology [41]. The morphological results in the present work suggest that this critical viscous balance was achieved, leading to the formation of homogeneous and structurally consistent fibres under the processing conditions employed. Polyurethane scaffolds exhibited a tensile strength of approximately 4.4 ± 0.3 MPa.

Porosity of the electrospun polyurethane (PU8038-HB-50) nanofiber scaffolds was also quantified using ImageJ software v1.54g from SEM image taken at magnification of × 4000. After calibrating the images using the embedded scale bar, the Otsu thresholding method [42] was applied to segment the fibrous network. In the resulting binary image, black pixels represented the fibres, while white pixels corresponded to pore spaces. Porosity was then calculated as the percentage of white pixels relative to the total number of pixels in the image, providing a direct measure of the void fraction within the scaffold. The binarized image used for the porosity analysis is available in the Supplementary Materials.

The analysis yielded porosity values of 49.6%. These results indicate a well-distributed fibrous structure across scales. This level of porosity is beneficial for applications requiring permeability and cellular infiltration, which are key requirements for applications in tissue engineering and regenerative medicine [43]. Moreover, the uniformity and continuity of the fibres suggest that the electrospinning conditions and polymer solution properties (notably the high molecular weight and resulting viscosity) were suitable to ensure both high porosity and mechanical integrity in the final scaffold.

### 3.6. Cytocompatiblity Evaluation of Polyurethane Films and Electrospun Meshes

To evaluate the suitability of biodegradable polyurethanes for biomedical applications, selected polyurethane samples (films and electrospun meshes) were assessed using the C166-GFP endothelial cell line. This autofluorescent cell model is known for its adherent behaviour, typically proliferating as monolayers on cytocompatible surfaces.

**Figure 6:**
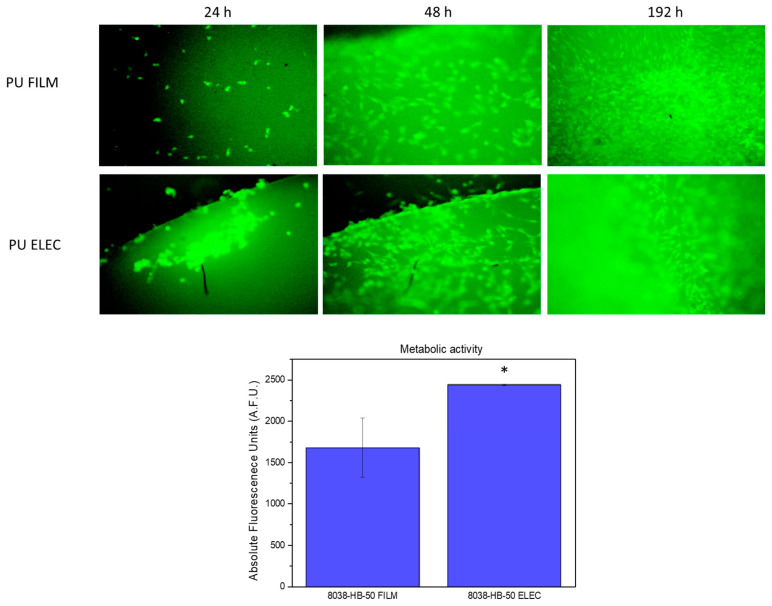
Up images of endothelial C166-GFP cell cultures after 24 h, 48 h, and 192 h on PU8038-HB-50 at 15× Down. Metabolic activity of cell culture at 192 h over samples (Alamar Blue). Significant differences stand for * (*p* ≤ 0.05).

The results demonstrated that both (film and electrospun) samples supported cell adhesion within 24 h post-seeding (Figure 6). At this time point, the cell line exhibited a rounded morphology on the polyurethane surfaces, which may indicate a slight delay in the initial adhesion process. However, at 48 h, the cell line displayed a well-spread elongated morphology on both the films and electrospun scaffolds, with clearly established focal adhesions, suggesting effective substrate integration and biocompatibility.

Then, cell monitoring over polyurethanes samples was extended to 192 h to evaluate a mid-term culture for total surface cell colonisation, and high-confluence areas were selected again in both surfaces. Finally, a quantitative analysis (Figure 6) of cell viability was performed using a metabolic activity test (Alamar Blue). Polyurethane films and meshes showed viable cell cultures over surfaces, with a statistically significant increase in the electrospun samples (*t*-student test) over the films.

The favourable biological performance observed for the PU samples—particularly the electrospun meshes—highlights their potential for applications in tissue engineering and regenerative medicine. Biodegradable polyurethanes can be used as temporary implants, not only in bone regeneration and orthopaedics but also across a range of medical fields, including cardiovascular devices, wound dressings, and sensors [10,44]. In these therapies, an appropriate degradation rate is essential, especially in paediatric and adolescent patients. The promising biological response exhibited by these newly synthesised PU materials therefore supports their potential for future development into tailored medical applications.

## 4. Conclusions

In this study, the influence of soft segment composition and length and chain extender type on the properties of biodegradable polyurethanes (PUs) was investigated, with a focus on their suitability for biomedical applications such as tissue engineering. Linear poly(ester-urethane)s were synthesised using CAPA^®^ polyols from the 8000 series, composed of random copolymers of ε-caprolactone and lactic acid with varying PLA content and molecular weights ranging from approximately 1000 to 3000 g/mol.

PUs exhibited high molecular weights (up to 255 kDa) and tuneable mechanical properties, with tensile strengths between 9 and 28 MPa and elongation at break ranging from 70% to over 1200%, depending on composition. Thermal and structural characterisation revealed that glass transition temperature (Tg) and crystallinity were strongly influenced by both the PLA content and the hard segment proportion, with higher PLA content and hard segment ratios resulting in increased Tg and reduced crystallinity of the hard segment.

In vitro degradation assays confirmed that PUs containing higher PLA content and hydrolysable chain extenders degraded more rapidly, offering a means to tailor degradation kinetics for specific clinical needs. The incorporation of lactic acid units supressed soft segment crystallinity and enhanced the hydrolytic sensitivity of the polymer chains. Furthermore, the use of the hydrolysable chain extender EDA-2CL promoted faster degradation of the hard segments compared to non-degradable alternatives such as 1,4-butanediol (BD).

Biocompatibility was confirmed using C166-GFP mouse endothelial cells, which adhered to and proliferated on both PU films and electrospun meshes. The nanofibrous architecture of the electrospun meshes, with fibre diameters ranging from 350 to 990 nm, closely resembled the structure of the extracellular matrix. This biomimetic morphology significantly enhanced cell viability and proliferation compared to the PU films. In conclusion, the CAPA^®^-based PUs developed in this work demonstrate promising features for biodegradable scaffold applications, combining mechanical adaptability, controlled degradation, and excellent cytocompatibility. These findings support the use of CAPA^®^ polyols as versatile building blocks for the development of tailored biomedical polyurethanes. Future research could explore in vivo performance and the incorporation of bioactive agents to further enhance tissue regeneration outcomes.

## Figures and Tables

**Table 1:** Molecular weight average (Mn), stress and strain at break of the polyurethanes studied.

Poly(ester-urethane)	Mn (HOPCLLOH)	Wt% L-Lactide	Mn (GPC) kDa	Stress@ Break (MPa)	Strain@ Break (%)
PU8015-HB-35	1017	14.7	105.5	9.0	532
PU8021-HB-35	1973	43.0	242.0	24.0	740
PU8025-HB-35	1970	18.0	99.8	16.0	668
PU8038-HB-35	2987	37.9	93.0	9.0	397
PU8015-HB-50	1017	14.7	38.3	12.7	99
PU8021-HB-50	1973	43.0	249.7	17.3	366
PU8025-HB-50	1970	18.0	36.7	13.8	74
PU8038-HB-50	2987	37.9	255.0	23.2	467
PU8015-HE-35	1017	14.7	168.5	19.7	1230
PU8021-HE-35	1973	43.0	162.6	27.8	992
PU8025-HE-35	1970	18.0	129.5	12.2	625
PU8038-HE-35	2987	37.9	113.9	21.2	919

**Table 2:** Temperature when the 5% mass loss occurs, and glass transition temperature (Tg), melting temperature (Tm), and enthalpy of fusion of each polyurethane (ΔH_f_).

Poly(ester-urethane)	Mn (HOPCLLOH)	Wt% L-Lactide	T^a^ (°C) 5% Weight	Tg (°C) DSC	Tm (°C) DSC	DH_f_ (J/g) DSC
PU8015-HB-35	1017	14.7	273.8	−36.8	115	19.4
PU8021-HB-35	1973	43.0	266.3	−11.8	135	19.1
PU8025-HB-35	1970	18.0	264.7	−43.9	154	22.7
PU8038-HB-35	2987	37.9	256.4	−29.3	157	23.6
PU8015-HB-50	1017	14.7	266.0	−32.8	145	29.4
PU8021-HB-50	1973	43.0	262.2	−11.8	151	24.0
PU8025-HB-50	1970	18.0	267.1	−46.9	157	38.6
PU8038-HB-50	2987	37.9	249.9	−30.6	166	38.4
PU8015-HE-35	1017	14.7	261.0	−41.4	162	16.6
PU8021-HE-35	1973	43.0	255.9	−13.7	187	17.2
PU8025-HE-35	1970	18.0	260.8	−47.2	186	17.6
PU8038-HE-35	2987	37.9	260.2	−29.2	188	23.6

**Table 3:** Weight variation (%) of the polyurethane samples after 7 and 21 days of immersion in phosphate-buffered saline (PBS), in the presence (+PLE) or absence (−PLE) of porcine liver esterase.

Poly(ester-urethane)	Weight Change (%)			
	7 days		21 days	
	PLE (−)	PLE (+)	PLE (−)	PLE (+)
PU8015-HB-35	0.77	−5.43	0.54	−0.80
PU8021-HB-35	0.67	−3.09	0.17	−5.25
PU8015-HB-50	0.29	−5.65	0.1	−0.64
PU8015-HE-35	0.54	−4.00	0.15	−6.52
PU8038-HE-35	0.37	−8.04	0.5	−12.2

## Data Availability

Data are contained within the article and Supplementary Materials.

## Supplementary Materials

The following supporting information can be downloaded at https://www.mdpi.com/article/10.3390/polym17121685/s1, Figure S1: ^1^H-NMR spectrum of CAPA^®^-8015 in deuterated chloroform. Figure S2: ^1^H-NMR spectrum of CAPA^®^-8021 in deuterated chloroform. Figure S3: ^1^H-NMR spectrum of CAPA^®^-8025 in deuterated chloroform. Figure S4: ^1^H-NMR spectrum of CAPA^®^-8038 in deuterated chloroform. Figure S5: Experimental stress–strain curves of polyurethanes with 35% HS content and butanediol (BD) as chain extender. Figure S6: Experimental stress–strain curves of polyurethanes with 50% HS content and butanediol (BD) as chain extender. Figure S7: Experimental stress–strain curves of polyurethanes with 35% HS content and EDA-2CL as chain extender. Figure S8: SEM image (×4000) and its corresponding binarized image of PU8038-HB-50 polyurethane used for porosity analysis. Table S1: Composition and molecular weight of CAPA® polyol.
