# Supplementary material for: Biodegradable Polyurethanes for Tissue Engineering: Influence of L-Lactide Content on Degradation and Mechanical Properties
Source: Polymers (Basel). 2025 Jun 17;17(12):1685. doi: 10.3390/polym17121685 (PMC12196973; doi:10.3390/polym17121685)
Supplement: Supplementary file 1 [file polymers-17-01685-s001.zip › polymers-3673341-supplementary.pdf]

Supporting Information

Biodegradable Polyurethanes for Tissue Engineering: Influence of L-Lactide Content on Degradation and Mechanical Properties.

Alejandra Rubio Hernández-Sampelayo,[1,2] Laura Diñeiro[1], Dulce María González-García[3], Enrique Martínez Campos[1], Rodrigo Navarro[1*] and Ángel Marcos-Fernández[1*]

1. Institute of Polymer Science and Technology (ICTP-CSIC) Juan de la Cierva 3, Madrid Spain.

2. Universidad Nacional de Educación a Distancia (UNED), Facultad de Ciencias, C/Bravo Murillo, 38, 28015 Madrid, Spain.

3. Instituto Politécnico Nacional, Escuela Superior de Ingeniería Química e Industrias Extractivas, UPALM-Zacatenco, Col Lindavista, Mexico City 07738, Mexico

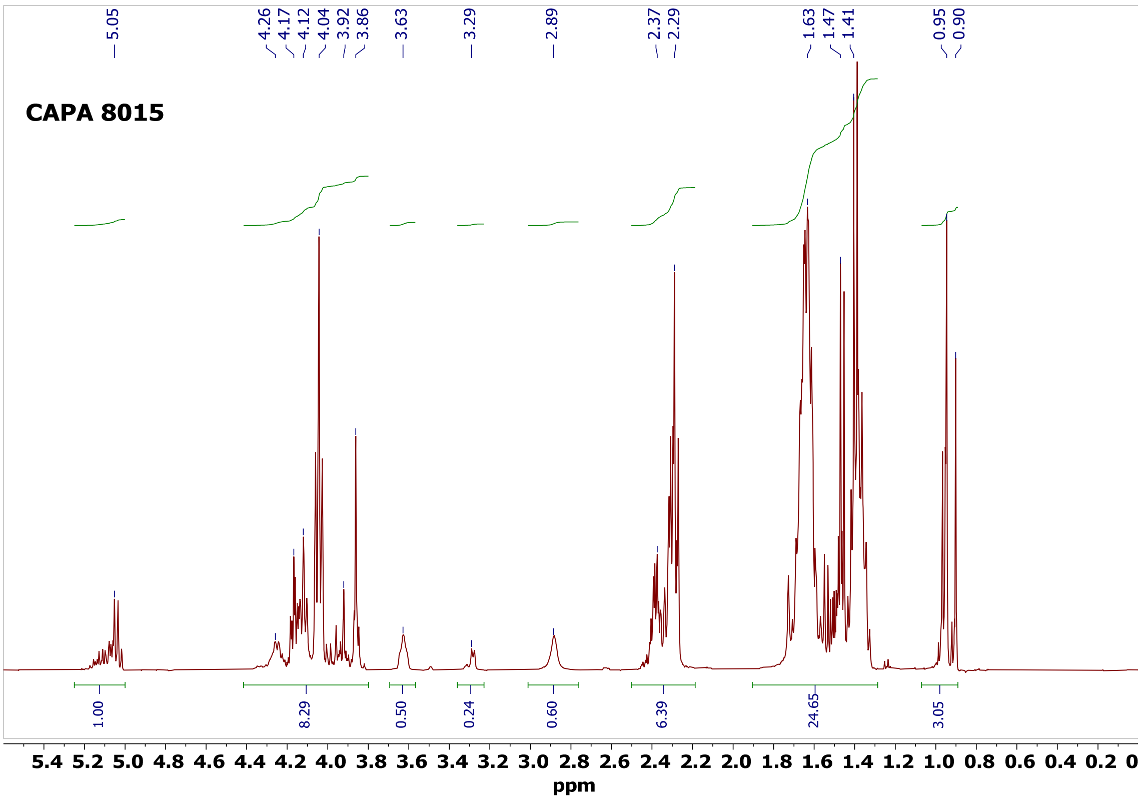

Figure S1: 1H-NMR spectrum of CAPA®-8015 in deuterated chloroform.

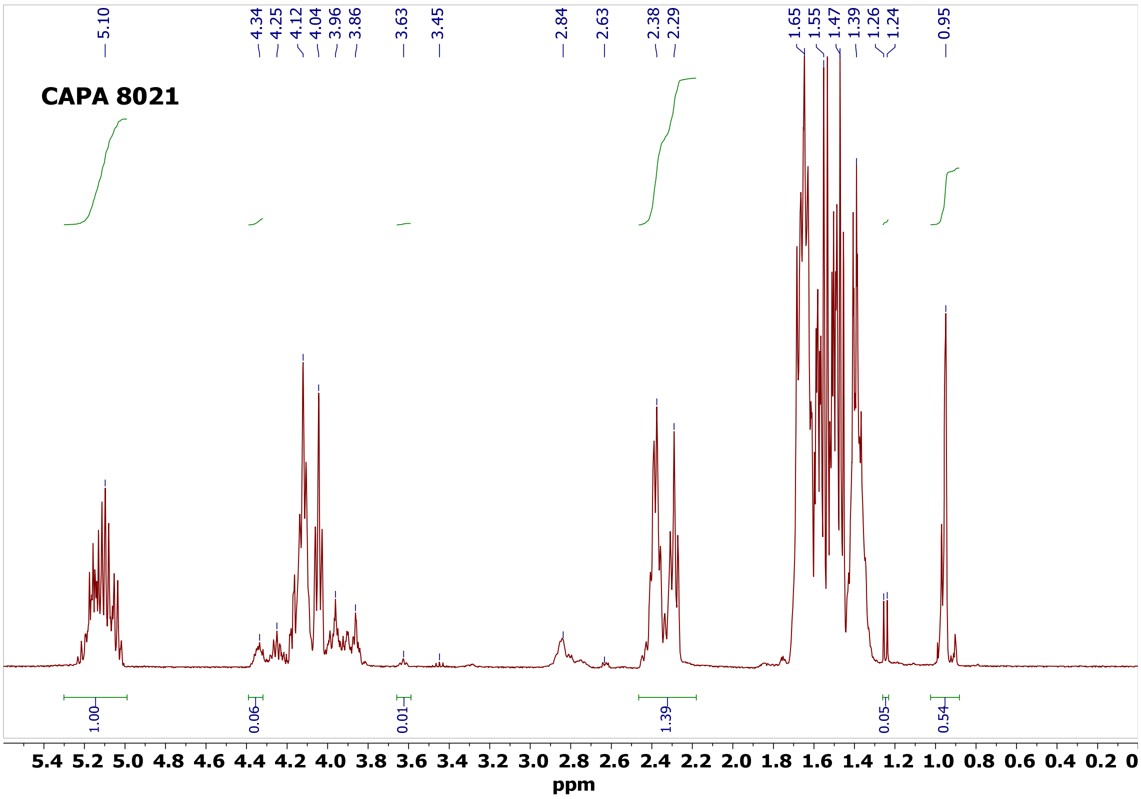

Figure S2: 1H-NMR spectrum of CAPA®-8021 in deuterated chloroform.

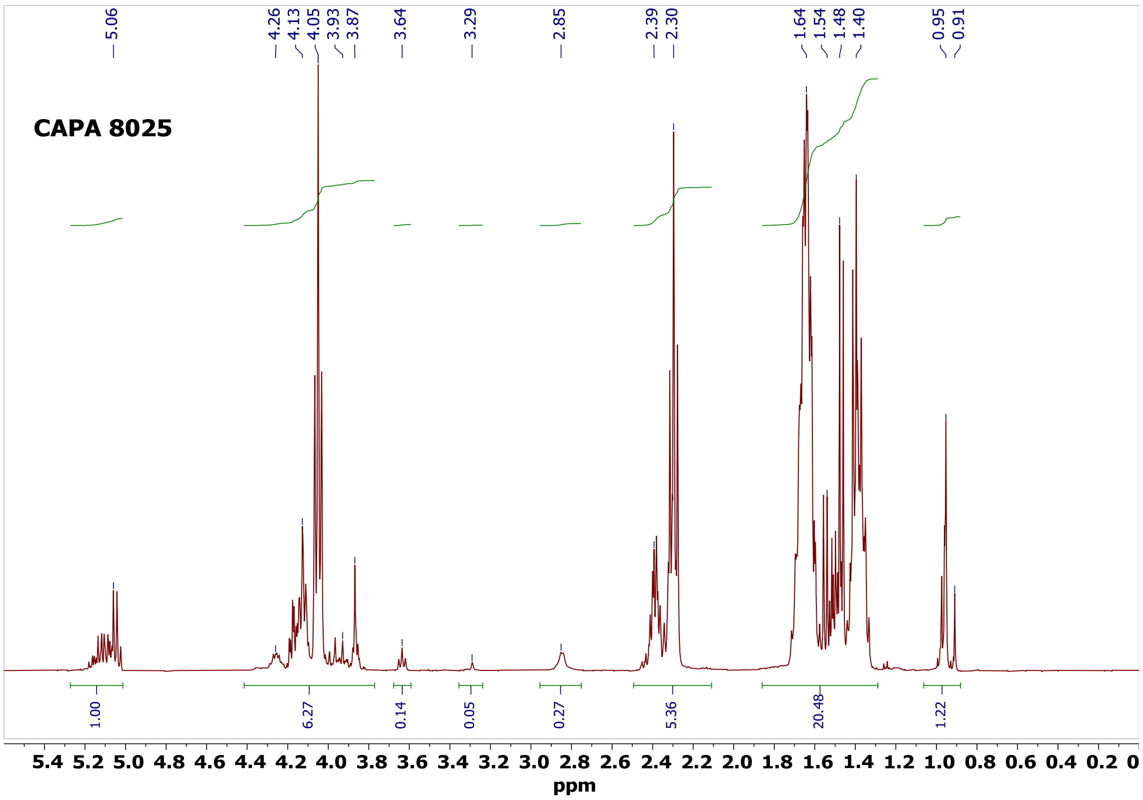

Figure S3: 1H-NMR spectrum of CAPA®-8025 in deuterated chloroform.

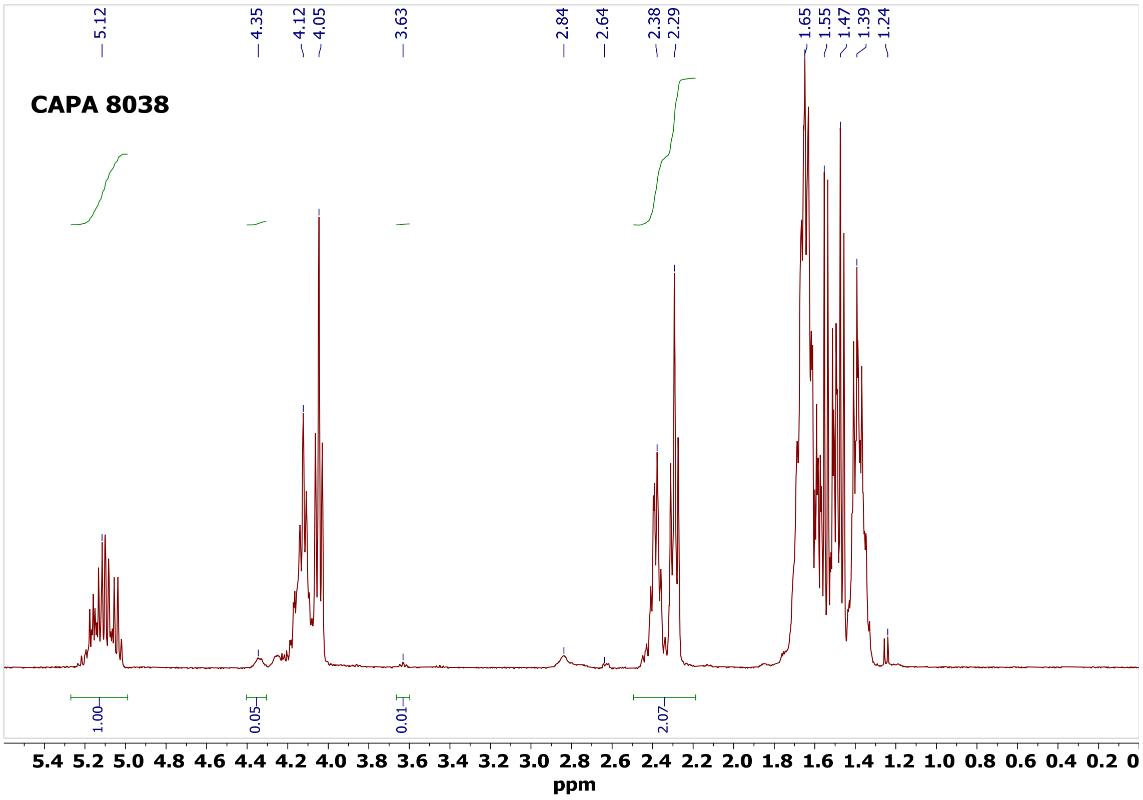

Figure S4: 1H-NMR spectrum of CAPA®-8038 in deuterated chloroform.

To determine the composition and molecular weight of each polyol, it is necessary to calculate the molar ratio between the signal areas at 5.02 ppm and 2.30 ppm, corresponding to lactide and caprolactone moieties, respectively. Finally, the molecular weight is calculated based on this molar ratio and the molecular weight of the initiator. All the data necessary for the determination of the molecular weight of the polyols are presented in the following table.

Table S1: Composition and molecular weight of CAPA® polyol.

**CAPA® Serie 8000**	**Initiator**	**Ratio CL/LA**	**% mol initiator**	**%mol CL**	**% mol LA**	**%wt LA**	**Mn CAPA®**
CAPA®8015	NPG	3.19	10.7	68.3	21.3	14.7	1017
CAPA®8021	BD	0.69	5.0	38.7	56.2	44.8	1973
CAPA®8025	NPG	2.68	5.2	69.1	25.7	18.1	1970
CAPA®8038	BD	1.03	3.1	48.1	48.8	37.9	2987

Where NPG is neopentylglycol and BD is 1,4-butanediol.

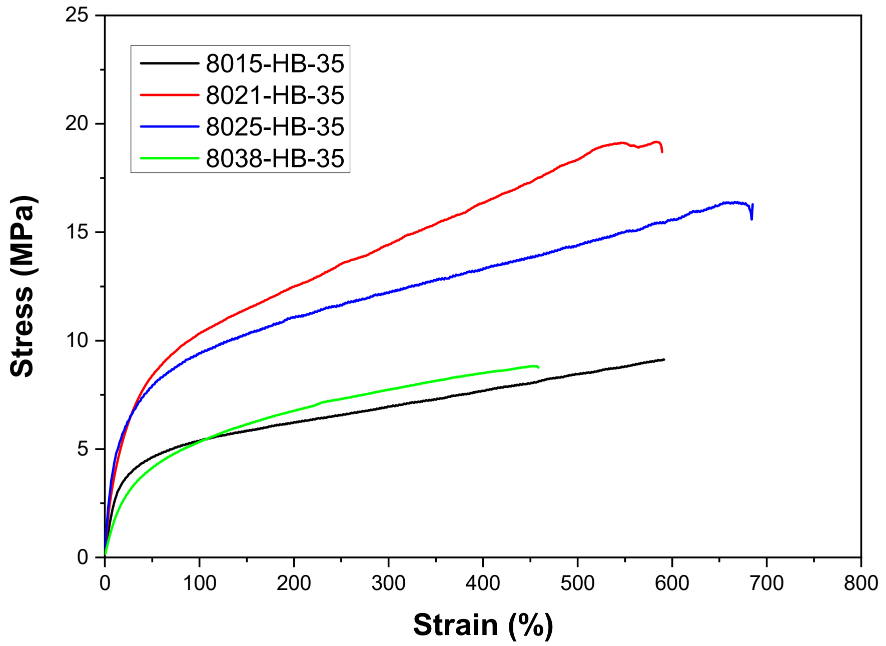

Figure S5: Experimental stress–strain curves of polyurethanes with 35% HS content and butanediol (BD) as chain extender.

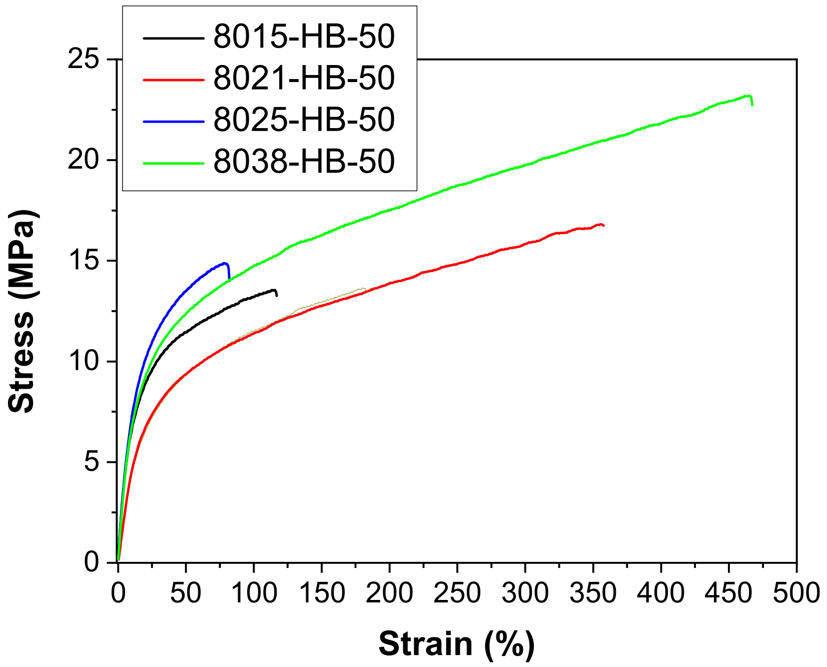

Figure S6: Experimental stress–strain curves of polyurethanes with 50% HS content and butanediol (BD) as chain extender.

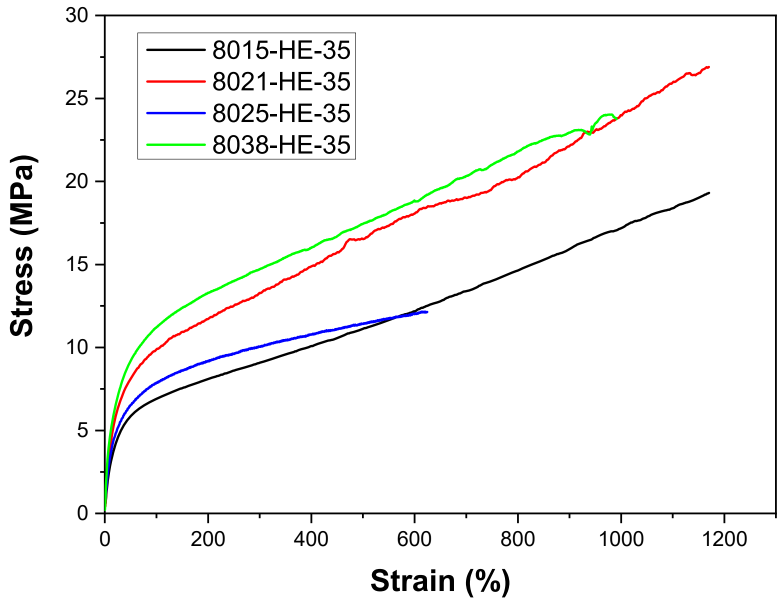

Figure S7: Experimental stress–strain curves of polyurethanes with 35% HS content and EDA-2CL as chain extender.

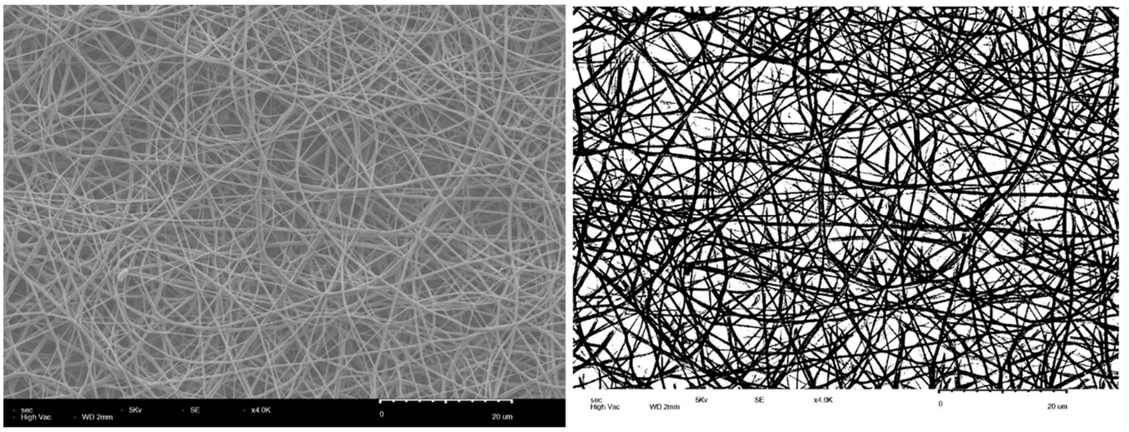

Figure S8: SEM image (×4000) and its corresponding binarized image of PU8038-HB-50 polyurethane used for porosity analysis.
